# Surveillance of *Plasmodium falciparum pfcrt* haplotypes in southwestern uganda by high‐resolution melt analysis

**DOI:** 10.1186/s12936-021-03657-7

**Published:** 2021-02-25

**Authors:** Kennedy Kassaza, Anna C. Long, Jennifer M. McDaniels, Mharlove Andre, Wasswa Fredrickson, Dan Nyehangane, Patrick Orikiriza, Darwin J. Operario, Joel Bazira, Juliet A. Mwanga-Amumpaire, Christopher C. Moore, Jennifer L. Guler, Yap Boum

**Affiliations:** 1 Epicentre Mbarara Research Centre, Mbarara, Uganda; 2 Department of Microbiology, Mbarara University of Science and Technology, Mbarara, Uganda; 3 Department of Biology, University of Virginia, Box 400328, 22904 Charlottesville, VA USA; 4 Division of Infectious Diseases and International Health, University of Virginia, Charlottesville, VA 22904 USA; 5 Department of Pediatrics and Child Health, Mbarara University of Science and Technology, Mbarara, Uganda

**Keywords:** Uganda, Chloroquine, *Plasmodium falciparum*, Resistance, *pfcrt*, HRM

## Abstract

**Background:**

Chloroquine (CQ) resistance is conferred by mutations in the *Plasmodium falciparum* CQ resistance transporter (*pfcrt*). Following CQ withdrawal for anti-malarial treatment, studies across malaria-endemic countries have shown a range of responses. In some areas, CQ sensitive parasites re-emerge, and in others, mutant haplotypes persist. Active surveillance of resistance mutations in clinical parasites is essential to inform treatment regimens; this effort requires fast, reliable, and cost-effective methods that work on a variety of sample types with reagents accessible in malaria-endemic countries.

**Methods:**

Quantitative PCR followed by High-Resolution Melt (HRM) analysis was performed in a field setting to assess *pfcrt* mutations in two groups of clinical samples from Southwestern Uganda. Group 1 samples (119 in total) were collected in 2010 as predominantly Giemsa-stained slides; Group 2 samples (125 in total) were collected in 2015 as blood spots on filter paper. The Rotor-Gene Q instrument was utilized to assess the impact of different PCR-HRM reagent mixes and the detection of mixed haplotypes present in the clinical samples. Finally, the prevalence of the wild type (CVMNK) and resistant *pfcrt* haplotypes (CVIET and SVMNT) was evaluated in this understudied Southwestern region of Uganda.

**Results:**

The sample source (i.e. Giemsa-stained slides or blood spots) and type of LCGreen-based reagent mixes did not impact the success of PCR-HRM. The detection limit of 10^− 5^ ng and the ability to identify mixed haplotypes as low as 10 % was similar to other HRM platforms. The CVIET haplotype predominated in the clinical samples (66 %, 162/244); however, there was a large regional variation between the sample groups (94 % CVIET in Group 1 and 44 % CVIET in Group 2).

**Conclusions:**

The HRM-based method exhibits the flexibility required to conduct reliable assessment of resistance alleles from various sample types generated during the clinical management of malaria. Large regional variations in CQ resistance haplotypes across Southwestern Uganda emphasizes the need for continued local parasite genotype assessment to inform anti-malarial treatment policies.

## Background

In 2018, the World Health Organization reported over 228 million cases of malaria across the world and 5 % of all cases are reported from Uganda [1]. The treatment of malaria is complicated on a global scale due to increased drug resistance to anti-malarials including chloroquine (CQ), sulfadoxine–pyrimethamine, mefloquine, and most recently, artemisinin [1]. CQ resistance is associated with mutations in the *P. falciparum* CQ resistance transporter (*pfcrt)* gene, which encodes a transmembrane protein localized to the parasite digestive vacuole membrane [2]. Mutations in residues 72–76 of *Pf*CRT contribute to altered efflux of CQ from the vacuole, which is the site of action for the drug [3]. The K76T mutation is a well-known marker of CQ resistance in Uganda [4]; this mutation may contribute to decreased sensitivity to lumefantrine [5]. While the normal haplotype across residues 72–76 is CVMNK, the mutant haplotype CVIET confers a high level of resistance [6] and is the most common in Africa [7]. The mutant SVMNT haplotype that is most common in South American isolates [6, 8–10] has a lower level of resistance compared to the CVIET haplotype [11]. This haplotype has recently been detected in Africa and surveillance of SVMNT is important due to possible cross-resistance to amodiaquine [12, 13].

The withdrawal of CQ as a therapy for malaria has led to the almost complete re-emergence of CQ-sensitive parasite populations in Malawi [14], Kenya and Tanzania [15, 16], and Northern regions of Uganda [5]. In other regions of the world, CQ resistant parasites persist decades after CQ cessation [17–19]. In general, a multitude of studies across Africa have observed a wide range in the proportion of CQ resistant parasites [20–28], which emphasizes the need for continued surveillance of resistance markers.

In this report, a PCR-based high-resolution melt (HRM) assay was used to screen 244 clinical samples from Southwestern Uganda for *pfcrt* mutant haplotypes. HRM is moderate throughput and yields few false positives [29, 30], exhibits high sensitivity and specificity in a variety of organisms [31–33], and can be employed inexpensively to screen samples prior to sequencing [30]. A previous highly accurate HRM assay of *pfcrt* haplotypes [34] was adopted for use on a distinct HRM instrument, evaluated for its performance with different reagents and sample types, and assessed for its ability to detect mixed alleles. Overall, both CVMNK and CVIET haplotypes were detected with high confidence in Southwestern Ugandan samples but resistance prevalence varied depending on location.

## Methods

### Study area and clinical isolates

Southwestern Uganda is mesoendemic for malaria. The highest transmission rates occur after the rainy season (September-January and March-May) [35]. Clinical samples used in this study were originally collected during previous cross-sectional studies in Southwestern Uganda. The studies were performed at Epicentre Mbarara Research Centre, a research arm of Médecins sans Frontières, and Mbarara University of Science and Technology [36, 37].

**Fig. 1:**
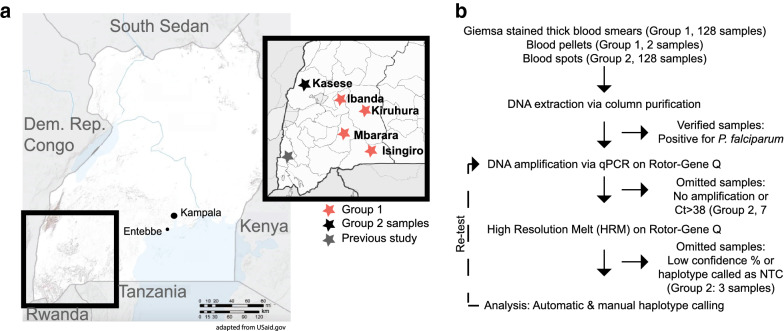
Clinical sample collection sites in Uganda and overview of methods for detection of ***pfcrt*** haplotypes.** a** Samples were collected in Southwest Uganda in the districts of Ibanda, Isingiro, Kiruhura, and Mbarara in 2010 (red stars) and the district of Kasese in 2015 (black star). Kunungu (grey star) was evaluated in previous studies [28, 40] and is used as a regional and temporal comparator. **b** Work flow of DNA isolation methods extracted from various sources followed by High-Resolution Melt analysis

Group 1 samples were collected while conducting household surveys of asymptomatic children < 5 years old from the districts of Ibanda, Isingiro, Kiruhura, and Mbarara in 2010 [32, 37–39] (Fig. 1a). Sampling was performed across low and high transmission seasons [37, 38]. Group 2 samples were collected from symptomatic patients in a peripheral health center in the district of Kasese across both low and high transmission seasons of 2015 [36] (Fig. 1a). All samples included in this study were positive for malaria, as confirmed by rapid diagnostic tests (RDT) and microscopy.

### Clinical sample preparation

Genomic DNA was extracted from Giemsa-stained slides, frozen blood pellets, and blood spots (summarized in Fig. 1b). Of the 119 total Group 1 samples, 117 DNA samples were from Giemsa-stained thick blood smears (stored at ambient temperature for 5 years before DNA purification) and two samples from frozen blood pellets (total volume of 100 µl) as previously reported [32]. Following extraction using the QIAamp DNA Mini Kit (Qiagen Inc., Germantown, Maryland, USA), these samples were also previously verified positive for *P. falciparum* using a species-specific HRM assay [32]. For Group 2, a total of 125 samples were extracted from filter paper blood spots using the QIAamp DNA Mini Kit (Qiagen Inc., Germantown, Maryland, USA). A subset of these samples were verified positive for *P. falciparum* as above prior to proceeding with haplotype analysis [32].

### Reference DNA and gene loci

*Plasmodium falciparum* genomic DNA controls were included in each run to validate assay performance and assign genotypes to the clinical samples. Genomic DNA from three *P. falciparum* reference strains with known *pfcrt* haplotypes (GenBank accession number NC_004328.2:458,600–461,695, gene ID: 2,655,199) were obtained from BEI resources (NIAID, NIH, Manassas, VA, USA): *P. falciparum* HB3 (wild type CVMNK haplotype, MRA-155G, contributed by Thomas E. Wellems), 7G8 (mutant haplotype SVMNT, MRA-152G, contributed by David Walliker), 7C424 (mutant haplotype CVIET, MRA-175G, contributed by Thomas E. Wellems). To ensure accurate genotyping, each control line was sequenced and confirmed for correct *pfcrt* mutations (Additional file 1: Fig. S1). Control samples provided reproducible melt curves which were used to determine the correct genotype of clinical samples with confidence.

### Quantitative PCR assays and cycling

The Rotor-Gene Q real-time PCR cycler (Qiagen Inc., Germantown, Maryland, USA) with a 72-well rotor was used for both PCR and HRM steps. Primers and probe with modified C3 spacer were purchased from Integrated DNA Technologies (IDT, Inc., Coralville, Iowa, USA). The primers and probe used were as follows [34]: Forward primer: 5′-GTAAAACGACGGCCAGTTTCTTGTCTTGGTAAATGTGCTCA-3′, Reverse primer: 3′-CAGGAAACAGCTATGACCGGATGTTACAAAACTATAGTTACCAAT-5′, HRM probe: 5′-GTGTATGTGTAATGAATAAAATTTTTG(3SpC3)-3′. Asymmetric PCRs were performed with the reverse primer in 10-fold excess to promote the accumulation of single-stranded DNA for probe binding [41]. The unlabelled HRM probe detected mutations across the 72–76 codon region. The C3 spacer on the end of the probe was necessary to prevent extension of the probe during PCR amplification. During the HRM steps, the probe disassociates from the mutant and wild type template DNA at distinct melting temperatures [34].

Either LightScanner Master mix (BioFire™ Defense, Salt Lake City, Utah, USA) or HotstarTaq Master mix (Qiagen Inc., Germantown, Maryland, USA) paired with 10× LCGreen Melting Dye (BioFire™ Defense, Salt Lake City, Utah, USA) was used for PCR amplification and HRM analysis. Although the Eva-Green-based Type-it HRM PCR kit (Qiagen Inc., Germantown, Maryland, USA) was recommended for use with the Rotor-Gene Q, The LightScanner and HotStarTaq mixes were selected based on their availability for purchase in Uganda.

Both mixes use LCGreen, which is a double strand DNA dye that is specifically designed for use during HRM since it facilitates the detection of heteroduplexes. The Lightscanner mix already has LCGreen included, while it must be added to the HotStar mix. In addition, the two mixes have slightly different levels of magnesium chloride, an important cofactor for the polymerase (Lighscanner: 2 mM, Hotstar: 3 mM) and employ different methods of preventing polymerase action prior to the start of PCR; this latter difference impacts the length of the polymerase activation step at the beginning of cycling (Lighscanner: antibody-based hot start requires 2 min, HotStar: inactive polymerase requires 15 min).

For LightScanner assays (Group 1 samples), the following components were added per 20µL reaction: 8µL of 2.5x LightScanner master mix, forward primer (1 µM final), reverse primer (10 µM final), probe (8 µM final), and 3 µL of sample DNA, reference DNA (1 ng) or nuclease-free water (no template control, NTC). For HotStarTaq assays (Group 2 samples), the following components were added per 20 µL reaction: 10µL of 2x HotStarTaq master mix, 1 µL of 10× LC Green, forward primer (1 µM final), reverse primer (10 µM final), probe (8 µM final), and 3µL of sample DNA, reference DNA (1 ng) or NTC.

**Fig. 2:**
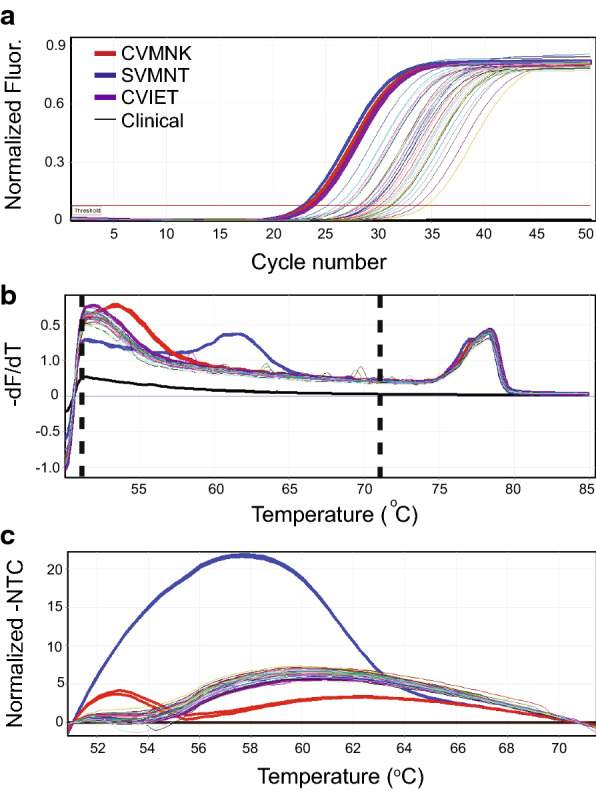
Summary of quantitative PCR and High-Resolution Melt parameters and analysis. Representative graphs of quantitative PCR and High-Resolution Melt analysis of clinical samples (thin coloured lines) and control samples (bold lines, blue: CVMNK wild type, HB3 parasite line; red: mutant SVMNT haplotype, 7G8 parasite line; purple: mutant CVIET haplotype, 7C424 parasite line). All analysis was performed with Rotor-Gene Q software and colours and line thickness were modified with TeeChart. **a** LCGreen fluorescence-based real time detection of DNA amplification of clinical samples and genomic DNA controls. Horizontal red line denotes the Ct threshold, which was determined for each assay. **b** A derivative plot displaying the melt analysis curve highlighting probe dissociation between normalization regions (denoted by dashed lines at 51 ^o^C and 71 ^o^C). The change in fluorescence was recorded in 0.2 ^o^C increments every 2 s. **c** Difference curve normalized to the no template control (NTC). The shape of the curve between the normalization region (see panel B) dictates the automatic genotype call

PCR cycling conditions were performed with an initial 2 min hold for LightScanner assays (15 min hold for HotStarTaq assays) at 95 °C followed by 50 cycles of 90 °C for 30 s, 60 °C for LightScanner assays (56 °C for HotStarTaq assays) for 30 s, and 72 °C for 30 s. Fluorescence data was acquired during the 72 °C step. The last cycle was followed by a 98 °C hold for 2 min and a 40 °C hold for 2 min before continuing to the HRM analysis (see Sect. 2.5). Fluorescence intensities of clinical samples, genomic DNA controls, and NTC were recorded in real-time throughout amplification cycles, which was used to determine the cycle threshold values for each assay (Fig. 2a).

### High‐resolution melt (HRM) parameters and analysis

Following PCR, the melting curve programme consisted of the following steps: a 90 s step of pre-melt conditioning at 50 °C followed by an increase in temperature from 50 to 90 °C in 0.2 °C increments every 2 s. The change in fluorescence was measured at each 0.2 °C increment. Manual gain optimization settings were as follows: set for 60 °C, HRM gain optimization was turned on at tube position 1, and set to select the highest fluorescence less than 70. Gain was set to a minimum and maximum reading of 1Fl and 3Fl, and a minimum and maximum gain of -10 and 10. Rotor-Gene Q Series Software version 2.2.1, Build 49 (Qiagen Inc., Germantown, Maryland, USA) was used for analysis of PCR and HRM data. Melt analysis curves were normalized between 51 and 71 °C (manually set) to capture the melting of the unlabelled probe from the full template (Fig. 2b). Narrower normalization regions were assessed and did not change the outcome of the analysis (Additional file 1: Fig. S2).

The haplotypes of clinical samples were automatically called by the Rotor-Gene Q software compared to the known genomic DNA controls, which produced reproducible HRM profiles. The difference curve (normalized to the NTC) was used to compare the melting profile of the probe/template duplexes (Fig. 2c). The confidence percentage threshold was set to 20 % to allow for low confidence calls to be recognized by the software. Confidence percentages were used as an integrity check (per the manufacturer’s instructions). Values above the threshold were subject to automatic calls predicted by the software. Values below the threshold were called as a “variation” and followed by a re-test (see Fig. 1b). If the amplitude or shape of the sample curve was different than the controls, the genotype was also determined by the programme to be a “variation”. For increased accuracy, each automatic call was manually inspected through visual comparison of the normalized difference curve (Fig. 2c). If a mistake in the genotype call was plainly discernible due to a visual discrepancy in the automatic genotype call and the difference curve, then a “manual call” was made (indicated in Additional file 1: Table S1). XY plots were generated using GraphPad Prism 7.0 (GraphPad Prism, La Jolla, CA).

### Data quality control

The combination of the Ct value, confidence percentage, and manual inspection allowed for heightened confidence in haplotype calling. Samples were re-tested if: (1) the Ct value was greater than 38 (equivalent to ~ 10^− 5^ng of parasite DNA), (2) the sample failed to amplify on the first run, or (3) an abnormal genotype needed to be verified. Of note, the majority of re-test samples gave consistent calls (Group 1: 12/14 (~ 86 %) and Group 2: 4/4 (100 %). Samples were omitted in three scenarios: (1) there was no amplification of the DNA and, therefore, no Ct value, (2) the Ct value of the samples were repeatedly above the 38 cycle cutoff, and (3) the genotype call was listed as NTC (no template control) (Fig. 1b). NTC melt curves were flat, displayed no Ct value, and did not resemble any of the positive controls.

## Results

### Various sample types and amplification reagents yield high confidence pfcrt haplotype calls

**Fig. 3:**
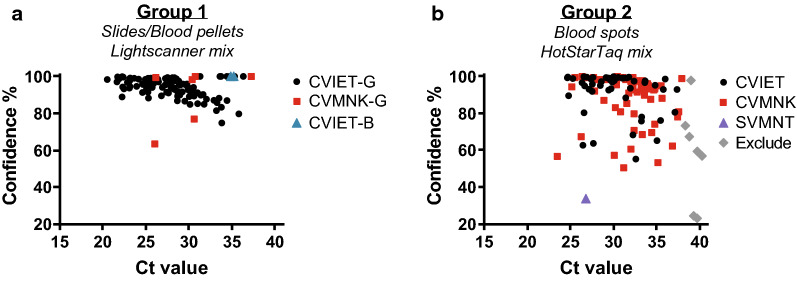
High-Resolution Melt analysis results from various sample sources and PCR-HRM mixes. Ct values (from quantitative PCR) and confidence percentages (from High-Resolution Melt analysis haplotype calls) display comparable results between Group 1 (**a**) and Group 2 (**b**) samples despite variable sources and PCR-HRM master mixes. Confidence percentages are automatically assigned by Rotor-Gene Q software to each sample according to their HRM melt profiles. Manual calls were set to a confidence percentage of 100 %. Samples with Ct values of > 38 cycles were excluded from the analysis (indicated as grey triangle).-G, Giemsa-stained blood slides; -B, blood pellets; CVMNK, wild type; SVMNT, mutant; CVIET, mutant

DNA from 244 samples was PCR-amplified and analysed using HRM on the Rotor-Gene Q instrument. In general, the range of Ct values for the quantitative PCR step was similar between the two groups (Fig. 3a and b). Mean Ct values of the Giemsa-stained slides and blood pellets (Group 1), and blood spot (Group 2) samples were 28.1, 35, and 31 cycles, respectively (Additional file 1: Table S1). Mean Ct values from CVMNK, CVIET and SVMNK haplotypes called in the two groups also displayed a similar range (Fig. 3a and b) and mean (mean Ct value of Group 1 CVMNK: 30.2; CVIET: 28.1 and Group 2 CVMNK: 31.6; CVIET: 30.2; SVMNT: 26.8 cycles). Two PCR-HRM reagent mixes were used over the course of the study due to differences in their accessibility: for Group 1 samples, LightScanner Master mix was used, which includes LC-Green dye;for Group 2 samples, HotstarTaq Master mix with added LC-Green dye was used. Similar to the analysis of sample type and haplotype, no notable differences were detected between the two different mixes in Ct value range (Fig. 3a and b) and mean (mean Ct value of Group 1: 28.2 and Group 2: 31.1 cycles).

Sample material was not normalized prior to amplification because DNA purified from clinical samples contains both human and parasite genomes. However, through the use of parasite DNA controls of known concentration (HB3, 7G8, 7C424), the clinical parasite DNA concentration range was estimated to be between ~ 1 ng and 10^− 5^ng per reaction. The lowest DNA concentration used in this study and the lowest DNA concentration detected by HRM were estimated to be between 10^− 4^ and 10^− 5^ng per reaction. This limit of detection for HRM is consistent with previous publications [34, 42, 43].

Following HRM steps, the *pfcrt* haplotype was automatically called by the Rotor-Gene software and inspected manually for accuracy (see Methods for details). Overall, mean confidence percentages across the different groups were > 89 % (Table 1; Fig. 3a and b). Further supporting the reliability of the automatic haplotype calls, there was an inverse correlation between Ct value and confidence percentage (p value of 2.243e−9, Additional file 1: Fig. S3). The average confidence percentage produced by automatic calls was > 90 % for all sample types and haplotypes (Table 1). Manual calls, which were made when a visual discrepancy was identified on the difference curve, represented only 9.2 % (11/244) of calls and were confined to Group 1 samples (Additional file 1: Table S1). Additionally, a larger percentage of samples from this group had to be re-tested due to high Ct values, NTC, or variant calls (10.9 % or 13/119 of Group 1 samples *versus* 3.2 % or 4/125 of Group 2 samples solely derived from blood spots, Additional file 1: Table S1). These Group 1 sample characteristics may be due to the slightly lower quality of DNA of the samples (derived from Giemsa-stained slides and collected 5 years earlier).

**Table 1:** Summary of High-Resolution Melt assay results and important metrics

Group	No. samples^a^	*pfcrt* haplotype		No. called (%)	Mean confidence %^d^	Mean Ct Value
1^b^	119	CVMNK	Wild type	6 (5.0) MC: 2	84.6	30.2 MC: 34.0
		SVMNT	Mutant	0 (0 %)	N/A	N/A
		CVIET	Mutant	112 (94.1) MC:11	93.8	28.1 MC: 34.4
		Unknown	Variation	1 (0.8)	n.d.	30.1
					**Overall Mean: 93.5 %**	**Overall Mean: 28.2**
2^c^	114	CVMNK	Wild type	60 (52.6)	88.2	31.6
		SVMNT	Mutant	1 (0.9 %)	33.6^e^	*26.8
		CVIET	Mutant	50 (43.9 %)	91.5	30.2
		Unknown	Variation	3 (2.6 %)	n.d.	35.1
					**Overall Mean: 89.7 %**	**Overall Mean: 31.1**

^a^Analyzed samples only include those with a Ct value < 38

^b^DNA samples were purified from predominantly Giemsa-stained slides

^c^DNA samples were purified from blood spots on filter paper

^d^Mean confidence % is assigned by software when automatic calls were made

^e^Denotes the exact value since an average could not be calculated; omitted from total average calculation

Underline denotes amino acid changes from the wild type CVMNK haplotype

*MC* Manual Calls, *CVMNK* Cys-Val-Met-Asn-Lys, *SVMNT* Ser-Val-Met-Asn-Thr, *CVIET* Cys-Val-Ile-Glu-Thr, *N/A* not applicable, *n.d.* not determined (a confidence value could not be assigned for these clinical samples due to their unknown melting profile)

### The mutant CVIET haplotype is common in southwestern Uganda

Overall, 66 % of the samples tested were called as the CVIET haplotype (162/244). The mutant CVIET haplotype was detected in the majority of the Group 1 samples (94.1 %; 112/119 samples, Table 1). Only 5 % (6/119) showed the wild type, CVMNK haplotype. However, the CVIET haplotype was less prominent in the Group 2 samples (43.8 %; 50/114, Table 1); more than half of samples were called as the CVMNK haplotype (52.6 %; 60/114). A single Group 2 sample displayed the mutant SVMNT haplotype (0.9 %; 1/114). However, due to a low confidence percentage (~ 34 %, Fig. 3b, Additional file 1: Table S1), classification of this sample was likely not accurate. Overall, only 4 clinical samples were called as variations (1/119 and 3/114 from Group 1 and 2 samples, respectively, Additional file 1: Table S1). Although the Ct value of these samples were in range of the other samples (~ 30–37 cycles), they exhibited an unknown melting profile that was not identified as CVMNK, SVMNK, or CVIET. These variants could, therefore, represent novel CQ mutations, alternative haplotypes (non-CVMNK/SVMNT/CVIET), or mixed haplotypes.

### The identification of mixed haplotypes requires manual inspection

**Fig. 4:**
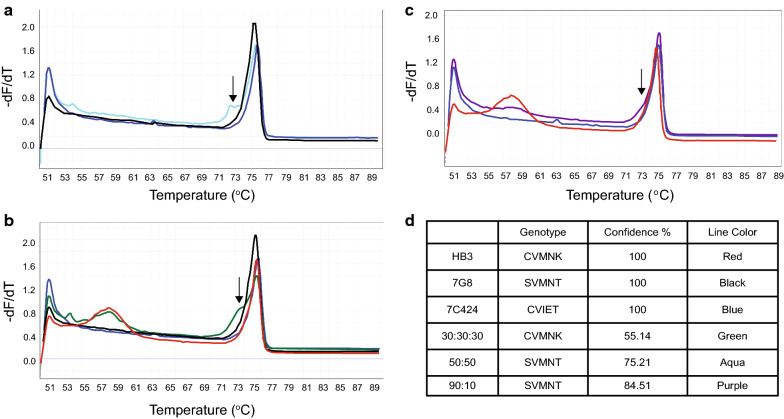
High-Resolution Melt analysis of mixed ***pfcrt*** haplotypes using the Rotor-Gene Q instrument. Derivative plots displaying the melt analysis curve for a 50:50 haplotype mixture of reference genomic DNA (**a**, SVMNT:CVIET), 30:30:30 mixture (**b**, CVMNK:SVMNT:CVIET), and 90:10 mixture (**c**, SVMNT:CVMNK). The heteroduplex ‘shoulder’ (arrow) is due to the formation and melting of a heteroduplex of both full length amplicon sequences. **d** Summary table of genotype, confidence % called by Rotor-Gene Q, and corresponding line colour

Other major haplotypes are not likely to be present in the clinical samples; wild type CVMNK and mutant CVIET haplotypes together covered 99.2 % of Group 1 samples and 96.5 % of Group 2 samples (Table 1). To investigate the ability of the Rotor-Gene Q instrument to call mixed haplotypes, control samples were tested at various ratios of 90:10 (SVMNT:CVMNK), 50:50 (SVMNT:CVIET), and 30:30:30 (CVMNK:SVMNT:CVIET). The sample with an equal proportion of two haplotypes (50:50) exhibited a prominent shoulder at a lower melting temperature (Fig. 4a). This shoulder was predicted to be due to heteroduplex formation (a mixture of both genotypes present) and was less prominent in non-equal mixtures (30:30:30 and 90:10, Fig. 4b and c). Notably, the Rotor-Gene Q software did not have the capability to automatically call the mixed haplotypes. As predicted, it called the predominant genotype with lower confidence (Fig. 4d). Given these findings, all samples were manually reviewed, including those with low confidence levels and variation calls; however, no samples exhibited the characteristic heteroduplex shoulder. Thus, mixed haplotypes were not likely the explanation for the variation calls. Using the heteroduplex shoulder, it was possible to manually identify mixtures of down to 10 % (Fig. 4c), as seen with other HRM platforms [42, 43]; analysis on the Rotor-Gene Q instrument was unable to reach a 1 % detection limit as reported with mutant allele amplification bias (MAAB) on a LightScanner-32 instrument [34].

## Discussion

Molecular genotyping studies have predominantly used gene-specific PCR followed by sequencing to identify resistance conferring mutations [26, 27, 44]. Other approaches such as ligase detection reaction-fluorescent microsphere assay [28, 40, 45] and the quantitative PCR-based malariaTAC [46] facilitate higher sample throughput and the parallel assessment of multiple resistance alleles. Although lower throughput than these methods, HRM-based assays allow for fast, reliable, and cost-effective haplotype analysis that is independent of sequencing and fluorescent probes [29, 30, 32, 34, 47]. Therefore, similar to SNP-loop mediated isothermal amplification (LAMP, [48–50], this method is amenable for surveillance efforts in settings in which malaria is endemic.

Importantly, the current studies show that HRM-based assessments of resistance alleles can be directly adopted for different instruments (i.e. BioFire Diagnostic’s Lightscanner as in Daniels et al. versus Qiagen’s Rotor-Gene Q used here). The flexibility of this method is emphasized by the high confidence data yielded from a variety of sample types and PCR-HRM reagents (Fig. 3). Giemsa-stained slides and blood spots are important sample sources that are collected during surveillance studies and are often archived for long periods of time. Using control samples, it is possible to detect mixed genotypes using the Rotor-Gene platform (Fig. 4); however, since the automatic genotype calls in the mixed samples had reduced confidence percentages, further evaluation would be required to investigate the exact genotypes present. The absence of mixed haplotypes in the clinical samples may be due to (1) the mesoendemic nature of infections in this region of Uganda, or (2) the possibility that minor alleles at this locus are below the HRM limit of detection (~ 10 % in this study). Due to the chance of missing minor resistance genotypes, this methodology may be better suited for surveillance (as conducted here) over ‘point of care’ assessments to determine anti-malarial treatment choice.

With the discovery of artemisinin resistance [51–53], the catalog of effective anti-malarials is dwindling. In response to growing resistance, there is a need to reevaluate historically effective treatments like CQ. Recent studies have assessed the response of parasite populations to CQ drug cessation in African countries [6, 8, 20–22, 25–27, 44, 54], but there has been limited research conducted in Southwestern Uganda. Resistance patterns in Tororo, located in Eastern Uganda, have been the most thoroughly documented [20, 40, 45, 55]; recent studies have also included few districts in Southwestern Uganda (i.e. Kanumgu and Kabale [28, 40]).

The prevalence of CQ resistance haplotypes was assessed in parasite populations from multiple sites across Southwestern Uganda (Ibanda, Isingiro, Kiruhura, and Mbarara collected in 2010, and Kasese collected in 2015). Dominance of the mutant CVIET haplotype in the samples, and lack of the mutant SVMNT haplotype (Table 1), is consistent with what has been observed in other areas of Uganda and throughout sub-Saharan Africa during a similar time frame [5, 8, 20, 21, 26, 27, 40, 44, 54]. Additionally, the prevalence of CVIET in Group 2 samples (~ 45 %, from Kasese, Table 1) is consistent with previous findings in the region (~ 50 %, from Kanungu in 2016, [28], see Fig. 1A for location comparisons). However, the high numbers of the mutant CVIET haplotype in Group 1 samples (~ 95 %, from Ibanda, Isingiro, Kiruhura, and Mbarara, Table 1) is well above what was previously measured in the region around a similar time period (< 5 %, from Kanungu in 2012, [40]). Despite limitations in the ability to assess temporal changes in CQ resistance levels across these regions (due to the collection of samples at two different time points, from different districts, and sampling methods), these studies illustrate that the variation in CQ resistance levels observed elsewhere also exists in locations across Southwestern Uganda. This finding emphasizes the need for continued regional assessments of resistant parasites profiles, using methods such as PCR-HRM, in order to inform any future use of CQ.

As an additional limitation, HRM-based methods cannot identify novel haplotypes because it measures the melting of a probe with a defined sequence from a homologous template. As mentioned above, there are not likely to be minor haplotypes in the study samples. Despite this result, it is possible that other minor resistance haplotypes have moved into the area (i.e. CVIEK, which has been detected in Sudan in 2000 [6] and in Nigeria in 2015 [56]). However, since the current study do not assess this haplotype, further investigations would be needed to explore this possibility in Uganda.

## Conclusions

In conclusion, HRM-based methods exhibit the flexibility required to conduct reliable assessment of resistance alleles from various sample types that may be generated during clinical management of malaria. Furthermore, regional variation in mutant *pfcrt* haplotypes across Southwestern Uganda is observed; this result emphasizes the need to continue local assessments of parasite genotypes to inform anti-malarial treatment policies.

## Supplementary Information

**Additional file 1: Table S1. **Summary of HighResolution Melt analysis on clinical samples. **Figure S1.** Targeted sequencingconfirms *pfcrt* haplotypes in control *Plasmodium* genomes. **Figure S2. **Change in normalization boundaries does notimpact automatic haplotype calling. **Figure S3. **Quantitative PCR Ct values are inverselyproportional to HRM confidence percentages.

## Data Availability

The datasets used and/or analysed during the current study are available from the corresponding author on reasonable request.

## Abbreviations

ACT: Artemisinin-based combination therapy
Ct: Cycle threshold
CQ: Chloroquine
HRM: High-resolution melt
MC: Manual calls
NTC: No template control
PfCRT: *Plasmodium falciparum* chloroquine resistance transporter
SW: Southwest
