# Supplementary material for: Surveillance of Plasmodium falciparum pfcrt haplotypes in southwestern uganda by high‐resolution melt analysis
Source: Malar J. 2021 Feb 25;20:114. doi: 10.1186/s12936-021-03657-7 (PMC7908690; doi:10.1186/s12936-021-03657-7)
Supplement: Supplementary file 1 — Additional file 1: Table S1. Summary of HighResolution Melt analysis on clinical samples. Figure S1. Targeted sequencingconfirms pfcrt haplotypes in control Plasmodium genomes. Figure S2. Change in normalization boundaries does notimpact automatic haplotype calling. Figure S3. Quantitative PCR Ct values are inverselyproportional to HRM confidence percentages. [file 12936_2021_3657_MOESM1_ESM.docx]

**Table S1. Summary of High Resolution Melt analysis on clinical samples.**

| **Group 1 Samples** | | | | | | | **Group 2 samples** | | | | | | |
| --- | --- | --- | --- | --- | --- | --- | --- | --- | --- | --- | --- | --- | --- |
| **Name** | Source | Mix | Ct | Hap. | Conf.% | Re-test | **Name** | Source | Mix | Ct | Hap. | Conf.% | Re-test |
| **3_12_2** | g | b | 28.66 | CVIET | 86.81 | N | **M010** | s | a | 33.23 | CVIET | 75.72 | N |
| **10_1_1** | g | b | 29.81 | CVIET | 94.61 | N | **M013** | s | a | 35.04 | CVIET | 65.11 | N |
| **10_16_2** | g | b | 29.93 | CVIET | 92.7 | N | **M033** | s | a | 31.42 | CVIET | 88.34 | N |
| **10_2_2** | g | b | 34.41 | CVIET | MC | N | **M038** | s | a | 32.26 | CVIET | 68.24 | N |
| **10_4_1** | g | b | 30.16 | CVIET | 93.5 | N | **M041** | s | a | 32.59 | CVIET | 55.1 | N |
| **10_9_2** | g | b | 29.34 | CVIET | 95.86 | N | **M057** | s | a | 29.85 | CVIET | 96.9 | N |
| **11_1_1** | g | b | 22.38 | CVIET | 99.6 | Y | **M087*** | s | a | 38.98 | CVIET | 97.8 | Y |
| **11_1_2** | g | b | 21.9 | CVIET | 98.74 | N | **M089** | s | a | 35.46 | CVIET | 76.06 | N |
| **11_12_1** | g | b | 30.41 | CVIET | 91.27 | Y | **M092** | s | a | 27.55 | CVIET | 95.78 | N |
| **11_14_1** | g | b | 23.1 | CVIET | 93.69 | N | **M094** | s | a | 29.44 | CVIET | 92.53 | N |
| **11_2_2** | g | b | 22.46 | CVIET | 93.3 | N | **M096** | s | a | 33.26 | CVIET | 77.92 | N |
| **11_3_1** | g | b | 27.26 | CVIET | 95.45 | N | **M098** | s | a | 26.57 | CVIET | 80.27 | N |
| **11_3_2** | g | b | 24.99 | CVIET | 96.26 | N | **M101** | s | a | 31.17 | CVIET | 99.07 | N |
| **11_6_1** | g | b | 24.61 | CVIET | 95.99 | N | **M111** | s | a | 31.02 | CVIET | 99.01 | N |
| **11_8_1** | g | b | 26.27 | CVIET | 97.63 | N | **M114** | s | a | 26.34 | CVIET | 96.56 | N |
| **13_1_1** | g | b | 33.82 | CVIET | 74.77 | Y | **M119** | s | a | 29.71 | CVIET | 93.3 | N |
| **13_3_1** | g | b | 30.05 | CVIET | 89.07 | N | **M124** | s | a | 28.58 | CVIET | 97.15 | N |
| **13_5_1** | g | b | 25.31 | CVIET | 88.28 | N | **M127** | s | a | 28.47 | CVIET | 95.03 | N |
| **13_8_1** | g | b | 25.07 | CVIET | 93.18 | N | **M128** | s | a | 37.37 | CVIET | 92.88 | N |
| **14_10_1** | g | b | 22.32 | CVIET | 94.26 | N | **M129** | s | a | 25.27 | CVIET | 98.81 | N |
| **14_8_1** | g | b | 30.54 | CVIET | 94.11 | N | **M134** | s | a | 26.44 | CVIET | 62.6 | N |
| **15_10_1** | g | b | 33.77 | CVIET | 83.25 | N | **M140** | s | a | 30.55 | CVIET | 99.41 | N |
| **15_11_1** | g | b | 23.18 | CVIET | 99.15 | N | **M158** | s | a | 26.89 | CVIET | 99.53 | N |
| **15_13_1** | g | b | 29.53 | CVIET | 86.56 | N | **M149** | s | a | 30 | CVIET | 99.83 | N |
| **17_13_1** | g | b | 29.08 | CVIET | 95.96 | N | **M162** | s | a | 35.78 | CVIET | 92.89 | N |
| **17_14_1** | g | b | 27.61 | CVIET | 98 | N | **M166** | s | a | 27.15 | CVIET | 93.02 | N |
| **17_15_1** | g | b | 32.04 | CVIET | 87.7 | N | **M168** | s | a | 32.39 | CVIET | 97.42 | N |
| **17_5_1** | g | b | 24.95 | CVIET | 98.38 | N | **M169** | s | a | 30.04 | CVIET | 99.5 | N |
| **17_8_1** | g | b | 28.54 | CVIET | 98.73 | N | **M172** | s | a | 27.37 | CVIET | 98.55 | N |
| **18_11_2** | g | b | 25.53 | CVIET | 97.51 | N | **M173** | s | a | 24.7 | CVIET | 99.63 | N |
| **18_11_3** | g | b | 32.9 | CVIET | 87.09 | N | **M180** | s | a | 26.65 | CVIET | 94.82 | N |
| **18_14_1** | g | b | 28.38 | CVIET | 97.71 | N | **M188** | s | a | 26.56 | CVIET | 99.82 | N |
| **18_5_1** | g | b | 28.94 | CVIET | 89.88 | N | **M189** | s | a | 27.52 | CVIET | 91.88 | N |
| **18_8_1** | g | b | 22.86 | CVIET | 96.8 | N | **M190** | s | a | 35.99 | CVIET | 98.54 | N |
| **19_12_2** | g | b | 30.99 | CVIET | 91.15 | N | **M191** | s | a | 29.46 | CVIET | 99 | N |
| **19_13_1** | g | b | 28.9 | CVIET | 96.61 | N | **M193** | s | a | 24.79 | CVIET | 89.52 | N |
| **19_2_1** | g | b | 28.31 | CVIET | 93.02 | N | **M194** | s | a | 37.12 | CVIET | 80.53 | N |
| **19_4_1** | g | b | 26.4 | CVIET | 98.48 | N | **M201** | s | a | 29.34 | CVIET | 99.05 | N |
| **19_8_1** | g | b | 33.73 | CVIET | MC | N | **M202** | s | a | 31.95 | CVIET | 93.55 | N |
| **2_11_1** | g | b | 27.63 | CVIET | 91.03 | N | **M204** | s | a | 33.63 | CVIET | 96.58 | N |
| **2_11_2** | g | b. | 28.82 | CVIET | 97.52 | N | **M205** | s | a | 24.64 | CVIET | 99.2 | N |
| **2_14_1** | g | b | 26.29 | CVIET | 98.56 | N | **M208** | s | a | 27.62 | CVIET | 99.42 | N |
| **2_2_1** | g | b | 27.94 | CVIET | 97.94 | N | **M209** | s | a | 30.09 | CVIET | 97.63 | N |
| **2_4_2** | g | b | 25.52 | CVIET | 95.16 | N | **M215** | s | a | 27.66 | CVIET | 63.64 | N |
| **2_7_1** | g | b | 34.75 | CVIET | MC | Y | **M182** | s | a | 31.78 | CVIET | 99.57 | N |
| **20_2_1** | g | b | 25.25 | CVIET | 93.67 | N | **M183** | s | a | 31.53 | CVIET | 99.14 | N |
| **20_7_2** | g | b | 24.16 | CVIET | 98.57 | N | **M007** | s | a | 29.14 | CVIET | 99.38 | N |
| **20_7_3** | g | b | 25.25 | CVIET | 91.51 | N | **M020** | s | a | 33.67 | CVIET | 97.52 | N |
| **21_1_1** | g | b | 31.3 | CVIET | MC | Y | **M027** | s | a | 29.64 | CVIET | 98.01 | N |
| **21_1_2** | g | b | 30.13 | CVIET | 84.82 | N | **M028** | s | a | 34.83 | CVIET | 95.14 | N |
| **21_4_7** | g | b | 25.26 | CVIET | 99 | N | **M029** | s | a | 30.02 | CVIET | 98.88 | N |
| **22_13_1** | g | b | 33.5 | CVIET | 88.2 | N | **M054*** | s | a | 39.71 | SVMNT | 23.02 | N |
| **r22_7_1** | g | b | 30.32 | CVIET | 90.96 | Y | **M077*** | s | a | 39.3 | SVMNT | 24.38 | N |
| **23_13_1** | g | b | 27.92 | CVIET | 93.84 | N | **rM140** | s | a | 26.79 | SVMNT | 33.64 | Y |
| **23_16_1** | g | b | 21.65 | CVIET | 97.02 | N | **M061** | s | a | 34.37 | Variation | N/A | N |
| **23_5_1** | g | b | 33.19 | CVIET | 81.43 | Y | **M065** | s | a | 36.94 | Variation | N/A | N |
| **23_6_2** | g | b | 26.12 | CVIET | 98.55 | N | **M082** | s | a | 33.96 | Variation | N/A | N |
| **23_9_1** | g | b | 31.64 | CVIET | 90.25 | N | **M053*** | s | a | n.d. | NTC | 99.23 | N |
| **24_11_1** | g | b | 28.09 | CVIET | 99.35 | N | **M056*** | s | a | n.d. | NTC | 98.68 | N |
| **24_2_3** | g | b | 27.44 | CVIET | 97.32 | N | **M074*** | s | a | n.d. | NTC | 97.28 | N |
| **24_2_4** | g | b | 26.39 | CVIET | 97.31 | N | **M034** | s | a | 36.86 | CVMNK | 62.08 | N |
| **24_4_1** | g | b | 21.73 | CVIET | 99.75 | Y | **M043** | s | a | 31.04 | CVMNK | 99.48 | N |
| **24_7_1** | g | b | 33.27 | CVIET | 85.77 | N | **M045** | s | a | 37.61 | CVMNK | 80.62 | N |
| **25_5_1** | g | b | 25.58 | CVIET | 99.07 | N | **M048** | s | a | 32.06 | CVMNK | 97.22 | N |
| **25_8_2** | g | b | 22.26 | CVIET | 99.91 | Y | **M058** | s | a | 35.57 | CVMNK | 87.99 | N |
| **27_1_1** | g | b | 35.3 | CVIET | MC | N | **M059** | s | a | 32.6 | CVMNK | 97.48 | N |
| **27_8_1** | g | b | 34.42 | CVIET | 86.96 | N | **M062** | s | a | 33.36 | CVMNK | 68.48 | N |
| **28_7_2** | g | b | 25.74 | CVIET | 94.03 | N | **M064** | s | a | 32.3 | CVMNK | 95.7 | N |
| **29_1_1** | g | b | 20.53 | CVIET | 98.33 | N | **M066** | s | a | 33.16 | CVMNK | 95.06 | N |
| **29_11_1** | g | b | 31.76 | CVIET | 84.92 | N | **M068** | s | a | 33.89 | CVMNK | 96.14 | N |
| **29_4_1** | g | b | 22.24 | CVIET | 88.87 | N | **M073** | s | a | 34.78 | CVMNK | 74.32 | N |
| **29_4_2** | g | b | 23.12 | CVIET | 98.79 | N | **M075** | s | a | 34.09 | CVMNK | 95.01 | N |
| **3_5_1** | g | b | 23.93 | CVIET | 94.64 | N | **M079** | s | a | 31.48 | CVMNK | 98.07 | N |
| **3_6_1** | g | b | 24.61 | CVIET | 91.34 | N | **M085** | s | a | 32.3 | CVMNK | 92.71 | N |
| **3_9_1** | g | b | 27.46 | CVIET | 97.48 | N | **M086** | s | a | 23.47 | CVMNK | 56.67 | Y |
| **32_13_18** | g | b | 23.16 | CVIET | 94.53 | N | **M095** | s | a | 34.82 | CVMNK | 93.16 | N |
| **33_13_1** | g | b | 29.24 | CVIET | 91.65 | N | **M099** | s | a | 33.95 | CVMNK | 94.81 | N |
| **34_15_1** | g | b | 30.44 | CVIET | 92.13 | N | **M100** | s | a | 32.36 | CVMNK | 97.28 | N |
| **r35_8_1** | P | b | 34.85 | CVIET | MC | N | **M106** | s | a | 25.53 | CVMNK | 99.47 | N |
| **37_4_1** | g | b | 26.47 | CVIET | 97.03 | N | **M109** | s | a | 28.85 | CVMNK | 86.94 | N |
| **4_10_1** | g | b | 23.65 | CVIET | 99.04 | N | **M113** | s | a | 30.47 | CVMNK | 99.8 | N |
| **4_11_1** | g | b | 30.59 | CVIET | 89.34 | N | **M115** | s | a | 35.46 | CVMNK | 94.69 | N |
| **4_6_1** | g | b | 31.38 | CVIET | 89.85 | N | **M116** | s | a | 31.98 | CVMNK | 60.53 | N |
| **r4_7_2** | P | b | 35.2 | CVIET | MC | N | **M121** | s | a | 32.35 | CVMNK | 70.77 | N |
| **4_7_3** | g | b | 27.71 | CVIET | 96.38 | N | **M122** | s | a | 37.89 | CVMNK | 98.51 | N |
| **46_10_3** | g | b | 28 | CVIET | 96.9 | N | **M125** | s | a | 25.1 | CVMNK | 93.99 | N |
| **46_4_1** | g | b | 25.74 | CVIET | 97.93 | N | **M126** | s | a | 33.81 | CVMNK | 96.27 | N |
| **53_8_1** | g | b | 28.29 | CVIET | 93.82 | N | **M130** | s | a | 32.22 | CVMNK | 99.23 | N |
| **55_1_2** | g | b | 27.57 | CVIET | 97.35 | Y | **M132** | s | a | 32.42 | CVMNK | 79.7 | N |
| **56_12_1** | g | b | 33.59 | CVIET | MC | N | **M133** | s | a | 34.4 | CVMNK | 69.69 | N |
| **56_12_2** | g | b | 31.01 | CVIET | 85.21 | N | **M141** | s | a | 33.2 | CVMNK | 92.68 | Y |
| **60_10_3** | g | b | 27.41 | CVIET | 91.44 | N | **M159** | s | a | 26.38 | CVMNK | 99.84 | N |
| **62_02_1** | g | b | 26.92 | CVIET | 94.09 | N | **M160** | s | a | 32.73 | CVMNK | 91.62 | N |
| **62_4_1** | g | b | 28.68 | CVIET | 89.34 | N | **M161** | s | a | 30.81 | CVMNK | 81.03 | N |
| **63_9_1** | g | b | 28.66 | CVIET | 91.94 | N | **M165** | s | a | 27.56 | CVMNK | 98.8 | N |
| **64_14_1** | g | b | 27.52 | CVIET | 99.41 | N | **M167** | s | a | 27.67 | CVMNK | 99.55 | N |
| **64_16_1** | g | b | 25.56 | CVIET | 95.25 | N | **M174** | s | a | 26.75 | CVMNK | 99.6 | N |
| **64_9_1** | g | b | 23.13 | CVIET | 97.59 | N | **M175** | s | a | 28.35 | CVMNK | 98.17 | N |
| **65_16_1** | g | b | 35.79 | CVIET | 79.7 | N | **M178** | s | a | 30.15 | CVMNK | 57.18 | N |
| **7_9_1** | g | b | 36.31 | CVIET | MC | Y | **M179** | s | a | 27.91 | CVMNK | 98.04 | N |
| **8_11_1** | g | b | 25.89 | CVIET | 96.07 | N | **M184** | s | a | 30.34 | CVMNK | 95.5 | N |
| **8_14_1** | g | b | 27.27 | CVIET | 98.86 | N | **M185** | s | a | 26.54 | CVMNK | 99.43 | N |
| **8_4_1** | g | b | 28.96 | CVIET | 95.27 | N | **M187** | s | a | 28.39 | CVMNK | 98.22 | N |
| **9_11_1** | g | b | 28.46 | CVIET | 97.73 | N | **M192** | s | a | 33.98 | CVMNK | 92.98 | N |
| **9_12_1** | g | b | 29.77 | CVIET | 95.87 | N | **M195** | s | a | 34.35 | CVMNK | 87.8 | N |
| **9_2_1** | g | b | 24.67 | CVIET | 95.61 | N | **M197** | s | a | 27.81 | CVMNK | 91.82 | N |
| **9_3_1** | g | b | 26.81 | CVIET | 89.47 | N | **M198** | s | a | 29.22 | CVMNK | 98.04 | N |
| **9_4_1** | g | b | 28.87 | CVIET | 95.61 | N | **M200** | s | a | 35.13 | CVMNK | 53.14 | N |
| **9_6_1** | g | b | 30.5 | CVIET | 92.1 | N | **M203** | s | a | 30.27 | CVMNK | 97.64 | N |
| **9_6_2** | g | b | 28.5 | CVIET | 98.32 | N | **M211** | s | a | 34.63 | CVMNK | 92.47 | N |
| **9_7_2** | g | b | 28.31 | CVIET | 99.33 | N | **M212** | s | a | 33.31 | CVMNK | 97.5 | N |
| **9_9_1** | g | b | 30.08 | CVIET | 92.32 | N | **M213** | s | a | 26.25 | CVMNK | 67.39 | N |
| **13_9_1** | g | b | 30.06 | Variation | N/A | Y | **M214** | s | a | 30.37 | CVMNK | 99.63 | N |
| **21_1_1** | g | b | 26.14 | CVMNK | 99.18 | N | **M016** | s | a | 37.4 | CVMNK | 77.98 | N |
| **27_1_2** | g | b | 26.03 | CVMNK | 63.62 | N | **M210** | s | a | 33.59 | CVMNK | 89.56 | N |
| **27_2_1** | g | b | 30.75 | CVMNK | MC | Y | **M002** | s | a | 31.54 | CVMNK | 85.22 | N |
| **44_5_4** | g | b | 37.3 | CVMNK | MC | N | **M021** | s | a | 30.22 | CVMNK | 83.11 | N |
| **48_11_1** | g | b | 30.57 | CVMNK | 77.1 | N | **M030** | s | a | 31.22 | CVMNK | 50.38 | N |
| **55_1_2** | g | b | 30.44 | CVMNK | 98.51 | N | **M031** | s | a | 31.97 | CVMNK | 97.43 | N |
|  |  |  |  |  |  |  | **M035** | s | a | 31.81 | CVMNK | 90.95 | N |
|  |  |  |  |  |  |  | **M199*** | s | a | 40.27 | CVMNK | 56.81 | N |
|  |  |  |  |  |  |  | **M083*** | s | a | 38.79 | CVMNK | 67.3 | N |
|  |  |  |  |  |  |  | **M069*** | s | a | 39.72 | CVMNK | 59.3 | N |
|  |  |  |  |  |  |  | **M060*** | s | a | 38.34 | CVMNK | 73.2 | N |
|  |  |  |  |  |  |  | **M012*** | s | a | 41.07 | CVMNK | 43.43 | N |

If Re-test=Y, the data from the repeated sample is shown here. For Groups 1 and 2, 12/14 (~86%) and 4/4 (100%) of samples gave consistent calls, respectively.

*Omitted samples due to Ct value >38 or no template control (NTC) call.

Sample sources: ^g^Giemsa-stained slides, ^p^blood pellets, ^s^blood spots.

PCR-HRM master mix: ^a^HotstarTaq reaction mix (from Qiagen), ^b^LightScanner reaction mix (from BioFire).

Hap, haplotype; Conf%, confidence percentage (automatically assigned by Rotor-Gene Q software to each sample according to their HRM melt profiles). CVMNK, wild type control; SVMNT, mutant; CVIET, mutant; MC, manual calls; N/A, not applicable

******Figure S1.** **Targeted sequencing confirms *pfcrt* haplotypes in control *Plasmodium* genomes.** A 300bp PCR product that includes amino acid residues 72-76 was sequenced to verify that the HRM genotyping call was accurate. **A.** The wild type CVMNK haplotype was confirmed by HB3 sequencing. **B.** The mutant SVMNT haplotype was confirmed by 7G8 sequencing. **C.** The mutant CVIET haplotype was confirmed by sequencing 7C424. Arrows denote nucleotide mutations that change amino acid sequences to C72S, M74I, N75E, K76T.

**Figure S2. Change in normalization boundaries does not impact automatic haplotype calling.** Difference curves of a subset of clinical samples with normalization regions set to 51-52^o^C and 70-71^o^C (**A**) or 51-52^o^C and 65-68^o^C (**C**). Genotyping calls of these samples are listed in **B** and **D**. NTC, no template control.

**
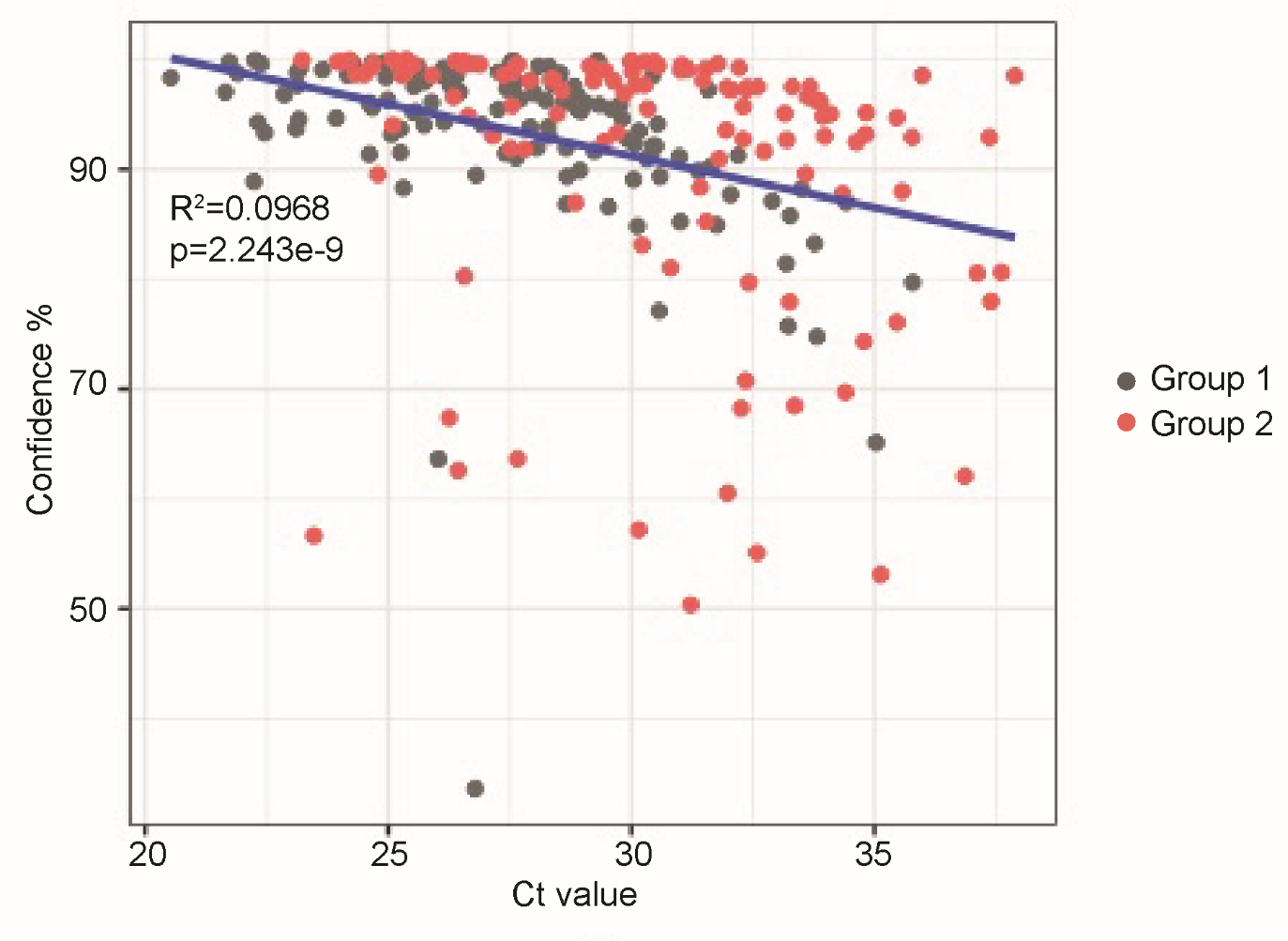
**

**Figure S3. Quantitative PCR Ct values are inversely proportional to HRM confidence percentages**. Following PCR-HRM, values from 244 clinical samples were plotted from Group 1 (grey) and Group 2 (orange). Blue line indicates trend line. Plot and statistical analysis were made using ggplot2 in RStudio.
